# Fasting blood glucose to HDL-C ratio as a novel predictor of clinical outcomes in non-diabetic patients after PCI

**DOI:** 10.1042/BSR20202797

**Published:** 2020-11-26

**Authors:** Qian-Qian Guo, Ying-Ying Zheng, Jun-Nan Tang, Ting-Ting Wu, Xu-Ming Yang, Zeng-Lei Zhang, Jian-Chao Zhang, Yi Yang, Xian-Geng Hou, Meng-Die Cheng, Feng-Hua Song, Zhi-Yu Liu, Kai Wang, Li-Zhu Jiang, Lei Fan, Xiao-Ting Yue, Yan Bai, Xin-Ya Dai, Ru-Jie Zheng, Xiang Xie, Jin-Ying Zhang

**Affiliations:** 1 Department of Cardiology, First Affiliated Hospital of Zhengzhou University, Zhengzhou 450052, P.R. China; 2 Key Laboratory of Cardiac Injury and Repair of Henan Province, Zhengzhou, China; 3 Department of Cardiology, First Affiliated Hospital of Xinjiang Medical University, Urumqi 830011, P.R. China; 4 Department of Cardiology, The First Affiliated Hospital, and College of Clinical Medicine of Henan University of Science and Technology, Luoyang 471003, China

**Keywords:** Coronary artery disease, Fasting Blood Glucose to HDL-C Ratio, Long-Term Adverse Outcomes, Percutaneous coronary intervention

## Abstract

**Background** The present study was to assess the prognostic value of fasting blood glucose to high-density lipoprotein cholesterol ratio (GHR) in non-diabetic patients with coronary artery disease (CAD) undergoing percutaneous coronary intervention (PCI).

**Methods and results** A total of 6645 non-diabetic patients from two independent cohorts, the CORFCHD-PCI study (*n*=4282) and the CORFCHD-ZZ (*n*=2363) study, were enrolled in Clinical Outcomes and Risk Factors of Patients with Coronary Heart Disease after PCI. Patients were divided into two groups according to the GHR value. The primary outcome included all-cause mortality (ACM) and cardiac mortality (CM). The average follow-up time was 36.51 ± 22.50 months. We found that there were significant differences between the two groups in the incidences of ACM (*P*=0.013) and CM (*P*=0.038). Multivariate Cox regression analysis revealed GHR as an independent prognostic factor for ACM. The incidence of ACM increased 1.284-times in patients in the higher GHR group (hazard ratio [HR]: 1.284 [95% confidence interval [CI]: 1.010–1.631], *P*<0.05). Kaplan–Meier survival analysis suggested that patients with high GHR value tended to have an increased accumulated risk of ACM. However, we did not find significant differences in the incidence of major adverse cardiac events, main/major adverse cardiovascular and cerebrovascular events (MACCE), stroke, recurrent myocardial infarction (MI) and bleeding events.

**Conclusions** The present study indicates that GHR index is an independent and novel predictor of ACM in non-diabetic CAD patients who underwent PCI.

## Background

Coronary artery disease (CAD) is one of the most common cardiovascular diseases (CVDs) in the world. Despite great advances achieved in the treatment of CAD, it still has high mortality. CAD is a complicated multifactor disease. Metabolic syndrome (MetS) is a major contributor to the epidemic of CAD [1], MetS includes abdominal obesity, high triglycerides (TGs), low high-density lipoprotein cholesterol (HDL-C), high blood pressure, and elevated fasting blood glucose (FBG) [2]. Muhlestein et al. found abnormalities of fasting glucose (FG) were much more prevalent (60%) than previously appreciated in patients with advanced CAD, they were associated with greater mortality risk than expected, despite elimination of ischemia by percutaneous coronary intervention (PCI) [3]. Dyslipidemia is one of the main risk factors for the development and progression of CVD, epidemiological studies strongly suggested that HDL-C levels were inversely associated with the risk of CAD [4]. Low HDL-C level is strongly associated with CVD development and CVD-related mortality [5]. Diabetes mellitus (DM) is well-established as a primary and secondary risk factor for CAD, it increases the risk of incident myocardial infarction (MI) and death in patients with established CAD [6]. However, there are relatively few studies investigating the prognosis values of FBG to HDL-C ratio (GHR) in non-diabetic patients with PCI. Therefore, our study examined the GHR index of non-diabetic patients with CAD undergoing PCI and discussed the effect of GHR index on long-term clinical outcomes.

## Methods

### Study design and population

**Figure 1:**
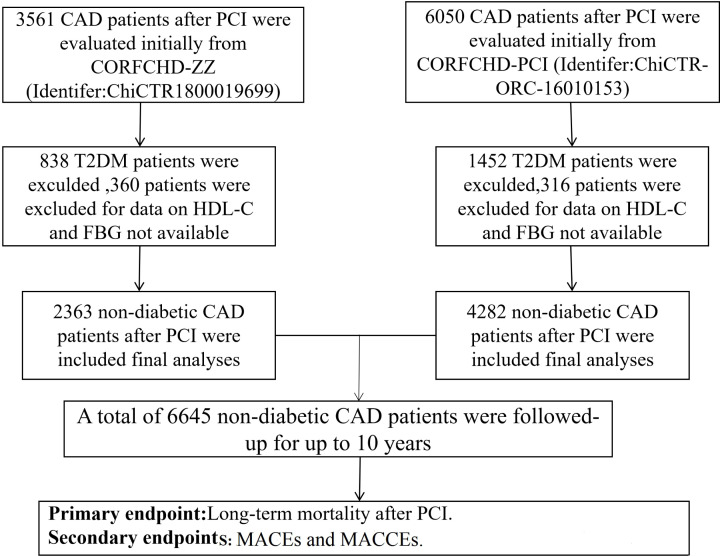
The flowchart of participants’ inclusion

CAD patients who underwent PCI at the First Affiliated Hospital of Zhengzhou University and First Affiliated Hospital of Xinjiang Medical University from January 2008 to December 2017 were included in the present study. DM patients and those with no HDL-C and FBG data were excluded from analysis. Finally, a total of 6645 non-diabetic CAD patients were enrolled in the present study (Figure 1). The details of the design are registered on http://www.chictr.org.cn (identifier: ChiCTR1800019699 and ChiCTR-ORC-16010153). The inclusion criteria were CAD patients including non-ST-segment elevation acute coronary syndrome (ACS), ST-segment elevation ACS and stable angina, who were undergoing coronary angiography, showing stenosis ≥ 70% and receiving at least one stent via implantation. We excluded patients who had DM, serious heart failure, rheumatic heart disease, valvular heart disease, congenital heart disease, pulmonary heart disease, and serious dysfunction of the liver or kidney. The present study complies with the Declaration of Helsinki and the protocol was approved by the ethics committee of the First Affiliated Hospital of Zhengzhou University and First Affiliated Hospital of Xinjiang Medical University. Because of the retrospective design of the study, the need to obtain informed consent from eligible patients was waived by the ethics committee.

### Definitions

Hypertension was defined as a systolic blood pressure (SBP) of ≥140 mmHg and/or a diastolic blood pressure (DBP) of ≥90 mmHg on at least three resting measurements on at least two separate healthcare visits according to the American Heart Association recommendations or with the use of any antihypertensive drug [7]. DM was defined as FBG ≥ 7.1 mmol/l or 2-h post-load glucose ≥ 11.1 mmol/l or current use of anti-diabetic medications [8]. Smoking and drinking status were defined as current tobacco and alcohol use.

### Clinical and demographic characteristics collection

Data on clinical and demographic characteristics, including age, sex, history of hypertension, family history of CAD, and smoking and drinking status, were collected from medical records. Imaging and laboratory data, including FBG, blood urea nitrogen (BUN), creatinine (Cr), uric acid (UA), lipid parameters and angiographic results were noted. During the follow-up period, the use of β-blockers, angiotensin-converting enzyme inhibitors (ACEIs), angiotensin II receptor blockers (ARBs), statins, aspirins, clopidogrels, ticagrelors and calcium channel blockers (CCBs) were recorded. GHR index was calculated by the formula [FBG (mmol/l)/HDL-C (mmol/l)].

### End points

The primary end point was the long-term mortality, including all-cause mortality (ACM) and cardiac mortality (CM), during the follow-up. The secondary end points were major adverse cardiovascular events (MACEs) defined as the combination of cardiac death, recurrent MI and target vessel reconstruction, and main/major adverse cardiac and cerebrovascular events (MACCEs) which have been described previously [9]. Briefly, deaths were considered a cardiac condition unless a non-cardiac definite cause was identified. Recurrent MI was defined as a new Q wave, and an increased concentration of creatine kinase MB to greater than five-times the upper limit of the normal range within 48 h after procedure or new Q waves or an increase in creatine kinase MB concentration to greater than the upper limit of the normal range plus ischemic symptoms or signs, if occurring more than 48 h after the procedure [9]. Stroke is defined as a sudden onset of vertigo, numbness, aphasia, or dysarthria caused by cerebrovascular disease, including hemorrhage, embolism, thrombosis, aneurysm rupture, and persisting for >24 h [9]. Bleeding events were determined according to the Bleeding Academic Research Consortium (BARC) standard [10]. Target vessel revascularization (TVR) was defined as any repetitive revascularization in a treated vessel where there was stenosis of at least 50% diameter in the presence of ischemic signs or symptoms or stenosis of at least 70% in the absence of ischemic signs or symptoms [9]. All incidents were determined by an adjudication committee that was blinded to the group of patients. All events were adjudicated by an event adjudication committee blinded to the group of the patients.

### Follow-up

In our study, the enrolled patients would receive regular follow-up after discharge by office visits or by telephone interview. And the patients were followed up for at least 2 years, and the longest follow-up time was 10 years. During the follow-up, the compliance of the drugs and adverse events were assessed by trained clinical physicians carefully.

### Statistical analyses

All analyses were performed using the SPSS 23.0 for Windows statistical software (SPSS Inc, Chicago, Illinois, United States). Continuous data are presented as the mean ± standard deviation. Categorical data are presented as the frequencies and percentages. The GHR was analyzed as a continuous variable categorized into two groups on the basis of the GHR cut-off value of 6.30. The cut-off value (6.30) is according to the analysis of the ROC curve for the baseline data of the study population. To compare parametric continuous variables, Student’s *t* tests were used, and to compare non-parametric continuous variables, Mann–Whitney U tests were used. Chi-squared (χ^2^) tests were used to compare categorical variables. Multivariate Cox regression analysis was used for determination of independent parameters for prognosis. Sequential models were developed to examine the incremental prognostic value of the parameters. Incremental factors added to the model at each step were considered significant when the difference in the log likelihood associated with each model corresponded to *P*<0.05. Long-term survival was analyzed using the Kaplan–Meier method. The *P*-values were two-sided, and *P*<0.05 was considered significant.

## Results

### Baseline characteristics

We divided the enrolled patients into two groups according to the the GHR value. Baseline characteristics of the two groups are shown in Table 1. There were 4543 patients in the low GHR group and 2102 patients in the high GHR group. Participants with higher GHR values were more likely to be male, with higher levels of Cr, UA, white blood count (WBC), heart rate (HR), FBG and TG, and higher incidence of smoking and alcohol drinking. Higher levels of total cholesterol (TC), low-density lipoprotein cholesterol (LDL-C) and SBP, higher use of β-blocker, statin and older patients were found in the low GHR group (all *P*-values <0.05). Meanwhile, family history, hypertension, levels of DBP, BUN and estimated glomerular filtration rate (eGFR), use of ACEI or angiotensin receptor antagonist (ARB) and CCB did not show significant difference between the two groups (all *P*-values >0.05).

**Table 1:** Baseline characteristics

Variables	GHR index		χ^2^ or T value	*P*
	Low (<6.30; *n*=4543)	High (≥6.30; *n*=2102)		
Age (years)	60.90 ± 10.98	59.47 ± 11.09	4.922	**<0.001**
Gender (male)	3284 (72.3%)	1621 (77.1%)	17.345	<**0.001**
Smoking	1664 (36.6%)	880 (41.9%)	16.682	<**0.001**
Alcohol drinking	1080 (23.8%)	620 (29.5%)	24.722	<**0.001**
Family history	308 (11.7%)	197 (11.9%)	0.020	0.884
HR (bpm)	74.29 ±16.20	75.50 ± 11.60	−3.086	**0.002**
Hypertension	1978 (43.5%)	871 (41.4%)	2.594	0.110
SBP (mmHg)	128.80 ± 18.56	127.39 ± 18.96	2.831	**0.005**
DBP (mmHg)	77.43 ± 11.41	77.05 ± 11.67	1.246	0.213
FBG (mmol/l)	4.90 ± 0.75	5.64 ± 0.82	−34.215	<**0.001**
BUN (mmol/l)	5.52 ± 2.86	5.64 ± 3.32	−1.457	0.145
Cr (mmol/l)	73.55 ± 22.94	76.96 ± 28.57	−4.785	<**0.001**
WBC (×10^9^)	7.01±2.27	7.52 ± 2.54	−8.197	<**0.001**
eGFR (%)	107.46 ± 199.78	102.46 ± 170.94	0.991	0.322
UA (mmol/l)	315.01 ± 86.45	321.21 ± 94.42	−32.130	<**0.001**
TG (mmol/l)	1.60 ± 1.00	2.16 ± 1.44	−18.212	<**0.001**
TC (mmol/l)	3.99 ± 1.07	3.83 ± 1.08	5.824	<**0.001**
LDL (mmol/l)	2.48 ± 0.90	2.37 ± 0.87	4.603	<**0.001**
CCB (*n*,%)	625 (13.8)	253 (12.1)	3.482	0.067
β-blocker (*n*,%)	2054 (45.3)	847 (40.5)	13.310	<**0.001**
ACEI or ARB (*n*,%)	1133 (25.0)	477 (22.8)	3.664	0.056
Statin (*n*,%)	2958 (65.3)	1184 (56.8)	44.349	<**0.001**

The bold P-Values are statistically different.

### Clinical outcomes

As shown in Table 2, for the primary end point, the incidence of ACM in the lower GHR group was 182 (4.0%) and that in the higher GHR group was 113 (5.4%). The difference was significant (*P*=0.013); also, the incidence of CM between the two groups showed a significant difference (2.9 vs. 3.9%, *P*=0.038). For the secondary end points, we found that there were no significant differences between the two groups in the incidence of MACEs (11.4 vs. 12.5%, *P*=0.205), MACCEs (13.4 vs. 14.2%, *P*=0.420). Besides, there were also no significant differences between the two groups in the incidence of stroke (2.3 vs. 1.9%, *P*=0.316), bleeding events (3.2 vs. 3.2%, *P*=1) or recurrent MI (2.9 vs. 2.7%, *P*=0.692).

**Table 2:** Clinical outcomes between two groups

Variables	GHR index		χ^2^ or T value	*P*
	Low (<6.30; *n*=4543)	High (≥6.30; *n*=2102)		
ACM (*n*,%)	182 (4.0)	113 (5.4)	6.355	**0.013**
CM (*n*,%)	134 (2.9)	83 (3.9)	4.540	**0.038**
MACEs (*n*,%)	519 (11.4)	263 (12.5)	1.638	0.205
MACCEs (*n*,%)	611 (13.4)	298 (14.2)	0.644	0.420
Stroke (*n*,%)	103 (2.3)	39 (1.9)	1.166	0.316
Bleeding (*n*,%)	83 (3.2)	53 (3.2)	0.004	1
Recurrent MI (*n*,%)	133 (2.9)	57 (2.7)	0.241	0.692

The bold P-Values are statistically different.

Univariate models for each of the predictor variables were created, and variables that were significant (*P*<0.05) in univariate Cox models were entered into multivariate Cox regression analysis. In multivariate Cox regression analysis, after adjusting for the traditional clinical prognostic factors and factors that were significant (*P*<0.05) in univariate Cox models, we found that GHR index could predict poor clinical outcomes. The GHR index was an independent predictor for ACM in the high GHR group.The incidence of ACM increased 1.284-times in patients in the high GHR group (hazard ratio [HR]: 1.284 [95% confidence interval [CI]: 1.010–1.631], *P*<0.05). (Table 3). However, after adjusting factors mentioned above in multivariate Cox regression analysis, the incidence of CM did not show significant diffrences between two groups (*P*>0.05) (Table 4).

**Table 3:** Cox regression analysis results for ACM

Variables	B	SE	Wald	*P*	HR	95% CI
Gender (male)	−0.060	0.155	0.152	0.696	0.941	0.695–1.275
Age (years)	0.044	0.006	57.393	<0.001	1.045	1.033–1.057
Smoking	−0.017	0.154	0.012	0.914	0.983	0.727–1.331
Alcohol drinking	0.057	0.166	0.117	0.732	1.058	0.765–1.465
Heart rate	0.023	0.005	23.229	<0.001	1.024	1.014–1.033
Cr	0.002	0.002	0.484	0.487	1.002	0.997–1.006
UA	0.001	0.001	2.194	0.139	1.001	1.000–1.002
WBC	0.089	0.023	14.751	<0.001	1.093	1.044–1.143
LDL-C	−0.132	0.069	3.629	0.057	0.877	0.76–1.004
GHR index high vs. low	0.250	0.122	4.180	0.041	1.284	1.010–1.631

Abbreviations: B, regression coefficient; SE, standard error.

**Table 4:** Cox regression analysis results for CM

Variables	B	SE	Wald	P	HR	95% CI
Gender (male)	−0.166	0.184	0.811	0.368	0.847	0.591–1.215
Age (years)	0.032	0.007	23.691	<0.001	1.033	1.019–1.046
Smoking	−0.071	0.178	0.159	0.690	0.931	0.657–1.321
Alcohol drinking	0.017	0.192	0.007	0.931	1.017	0.698–1.481
Heart rate	0.026	0.006	20.892	<0.001	1.026	1.015–1.037
Cr	0.002	0.002	0.605	0.437	1.002	0.997–1.006
UA	0.001	0.001	3.163	0.075	1.001	1.000–1.003
WBC	0.068	0.028	6.146	0.013	1.071	1.014–1.130
LDL-C	−0.113	0.080	2.006	0.157	0.893	0.763–1.045
GHR index high vs. low	0.222	0.143	2.415	0.120	1.248	0.944–1.651

Abbreviations: B, regression coefficient; SE, standard error.

**Figure 2:**
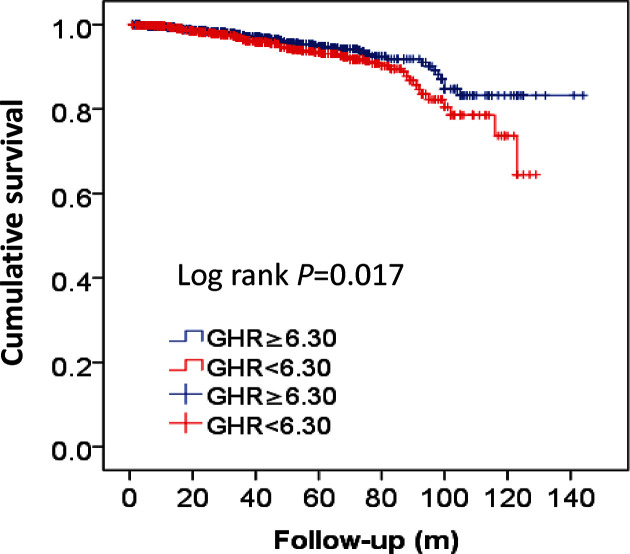
Kaplan–Meier curves for survival analysis of ACM-free survival

Kaplan–Meier survival analysis suggested that patients with high GHR value exhibited increased accumulated risk of ACM (Log-rank *P*=0.017; Figure 2).

## Discussion

The present study investigated the prognostic value of GHR in non-diabetic CAD patients with PCI and found that GHR index was an independent poor prognosis factor at levels ≥6.30. High levels increased long-term ACM 1.284-times. To reduce the risk of confounding factors, we adjusted a comprehensive list of characteristics that influence the risk of cardiovascular events to examine the association between GHR value and clinical outcomes.

Although a large number of novel biomarkers, such as genetic variation [11], miRNA [12] and LncRNA [13] have emerged in recent years, their predictive performances on the outcomes of CAD patients after PCI are limited and performing detection methods for these biomarkers are expensive. Recently, the prognostic role of hematologic biomarkers in patients with CVD has been recognized, such as RDW [14], GPR [15], TG/HDL-C ratio [16], IFG [17], TyG [18], MPV [19], which are efficient biomarkers for predicting clinical outcomes in patients with CAD.

However, there are few researches that focus on non-diabetic CAD patients. In order to find a reliable biomarker to predict the outcome of CVD in non-diabetic CAD patients. Our study first investigated the relation between the GHR value and outcomes in non-diabetic CAD patients after PCI. Since hyperglycemia and low level of HDL-C are well-known risk factors of CVD, the predictive value of FBG and HDL-C on cardiovascular outcomes is less effective. HDL-C plays a protective role in CVDs by enhancing cholesterol metabolism [20], while plasma glucose might be associated with dysfunction of endothelial cells and platelets [21–23]. Their values in predicting CVD risk might be better interpreted when they were considered as a whole.

In the present study, we recruited 6645 non-diabetic CAD patients who underwent PCI. We divided these patients into two groups according to GHR value, we found that patients in the higher GHR group had higher incidence of ACM. Several baseline characteristic variables showed significant differences between the two groups, such as gender, smoking, age, alcohol drinking, Cr, UA, WBC, TC, LDL-C. Considering the impact of these confounding factors and some traditional clinical prognostic factors, we performed multivariable Cox regression analyses. After adjusting for these confounders, the GHR index remained an independent predictor of ACM in non-diabetic CAD patients after PCI. Therefore, the results are credible and cannot be accidental.

The elevated ACM in the higher GHR group can be explained as follows: (1) Dyslipidemia is a major risk factor for atherosclerotic CVD, it includes hypertriglyceridemia, hypercholesterolemia, low high-density lipoprotein levels, high low-density lipoprotein levels etc [24]. HDL-C level is inversely associated with CVD and coronary death, independently from other traditional risk factors [25], HDL-C exerts its cardioprotective effects mainly through its role in reverse cholesterol transport (RCT), including removing cholesterol from peripheral cells in the arterial wall and transporting it to the liver for recycling [26,27]. (2) Hyperglycemia is also a traditional risk factor for CVD [3]. Previous studies have shown that appropriate glycemic control can provide long-term cardiovascular benefits [28,29]. Glucose itself may be atherogenic by increasing oxidative stress [30,31] and vascular inflammation, non-enzymatically glycating low-density liprotein, other apolipoproteins, and clotting factors [32] and facilitating the formation of advanced glycation end-products in the vessel wall and matrix [33]. (3) Low HDL-C level and high FBG level can accelerate the evolution of coronary atherosclerosis in CAD patients, so elevated ACM and CM in higher GHR group can be credible.

Our study had several limitations. First, this is an observational study with potential selection bias. Second, the sample size was small, and the clinical follow-up duration was not long enough, which might influence the reliability of results. Third, laboratory parameters were only measured once at admission with a potential bias due to measurement error. Finally, residual compounding factors and unrecorded variables might also affect outcomes.

## Conclusion

The present study shows that the baseline GHR index is a simple, reliable, cheap and independent predictor of adverse outcomes in non-diabetic CAD patients who underwent PCI. The GHR index deserves further validation in other populations.

## Data Availability

The data will not be shared, because the identified participant information is included in the data.

## Abbreviations

ACEI: angiotensin-converting enzyme inhibitor
ACM: all-cause mortality
ACS: acute coronary syndrome
ARB: angiotensin II receptor blocker
BUN: blood urea nitrogen
CAD: coronary artery disease
CCB: calcium channel blocker
CM: cardiac mortality
Cr: creatinine
CVD: cardiovascular disease
DBP: diastolic blood pressure
DM: diabetes mellitus
FBG: fasting blood glucose
GHR: fasting blood glucose to high-density lipoprotein cholesterol ratio
HDL-C: high-density lipoprotein cholesterol
HR: hazard ratio
LDL-C: low-density lipoprotein cholesterol
MACCE: main/major adverse cardiovascular and cerebrovascular event
MACE: major adverse cardiovascular event
MetS: metabolic syndrome
MI: myocardial infarction
PCI: percutaneous coronary intervention
ROC: receiver operating characteristic curve
SBP: systolic blood pressure
TC: total cholesterol
TG: triglyceride
UA: uric acid
WBC: white blood count
